# Resistive Switching Phenomenon Observed in Self-Assembled Films of Flame-Formed Carbon-TiO_2_ Nanoparticles

**DOI:** 10.3390/ma14164672

**Published:** 2021-08-19

**Authors:** Mario Commodo, Gianluigi De Falco, Ettore Sarnelli, Marcello Campajola, Alberto Aloisio, Andrea D’Anna, Patrizia Minutolo

**Affiliations:** 1 Istituto di Scienze e Tecnologie per l’Energia e la Mobilità Sostenibili, STEMS-CNR, P.le Tecchio 80, 80125 Napoli, Italy; mario.commodo@stems.cnr.it; 2 Dipartimento di Ingegneria Chimica, dei Materiali e della Produzione Industriale, Università degli Studi di Napoli Federico II, P.le Tecchio 80, 80125 Napoli, Italy; gianluigi.defalco@unina.it; 3 Institute for Superconductors, Innovative Materials and Devices, CNR-SPIN, S.S. di Napoli, Via Campi Flegrei 34, 80078 Pozzuoli, Italy; ettore.sarnelli@spin.cnr.it (E.S.); aloisio@na.infn.it (A.A.); 4 INFN—Sezione di Napoli, Via Cintia, 80126 Napoli, Italy; macampajola@na.infn.it; 5 Dipartimento di Fisica “E. Pancini”—Università degli Studi di Napoli Federico II, Via Cintia, 80126 Napoli, Italy

**Keywords:** bipolar switching resistance, nanostructured thin films, pinched hysteresis loop, flame synthesis, carbon nanoparticles, TiO_2_ nanoparticles

## Abstract

Nanostructured films of carbon and TiO_2_ nanoparticles have been produced by means of a simple two-step procedure based on flame synthesis and thermophoretic deposition. At first, a granular carbon film is produced on silicon substrates by the self-assembling of thermophoretically sampled carbon nanoparticles (CNPs) with diameters of the order of 15 nm. Then, the composite film is obtained by the subsequent thermophoretic deposition of smaller TiO_2_ nanoparticles (diameters of the order of 2.5 nm), which deposit on the surface and intercalate between the carbon grains by diffusion within the pores. A bipolar resistive switching behavior is observed in the composite film of CNP-TiO_2_. A pinched hysteresis loop is measured with SET and RESET between low resistance and high resistance states occurring for the electric field of 1.35 × 10^4^ V/cm and 1.5 × 10^4^ V/cm, respectively. CNP-TiO_2_ film produced by flame synthesis is initially in the low resistive state and it does not require an electroforming step. The resistance switching phenomenon is attributed to the formation/rupture of conductive filaments through space charge mechanism in the TiO_2_ nanoparticles, which facilitate/hinder the electrical conduction between carbon grains. Our findings demonstrate that films made of flame-formed CNP-TiO_2_ nanoparticles are promising candidates for resistive switching components.

## 1. Introduction

Flame synthesis is a very attractive method for the production of nanomaterials, being relatively inexpensive, scalable and based on a single-step process. While combustion is a longstanding and well-established method for production of carbon materials, such as carbon black, its use in the production of new functional nanomaterial is more recent and it is continuously gaining ground. It makes available new kinds of nanoparticles, such as mixed oxides, metal salts and even pure metals, which are produced with fine and controlled characteristics [1,2]. Flame synthesis, indeed, allows controlling precisely particle size, crystallinity, and phase purity. This is particularly attractive in the case of TiO_2_, whose properties are strongly influenced by these parameters. In recent years, the interest toward TiO_2_ nanoparticle production has increased due to its natural abundance, non-toxicity, and low cost. Its technological exploitation is thus broadly expanding: TiO_2_ finds applications in catalysis [3], in gas sensors [4], as protective coating due to its anti-bacterial and anticorrosive properties [5,6,7], as anode materials in lithium-ion batteries [8], in solar cell and electronic devices [9,10,11,12], and more. A strategy to improve the device performance is to produce carbon/TiO_2_ composites, which are beneficial, for instance, for photocatalytic activity [13] and anodic performance in lithium-ion batteries [14]. Among the conductive carbon material used in composite, various kinds of carbon structures are being explored [15,16,17,18] including graphene oxide, nanowires and nanotubes. Furthermore, research on TiO_2_ nanoparticles embedded in amorphous carbon is ongoing [19]. 

Interfaces between TiO_2_ and metals are also the basis of one of the more intriguing effects observed in TiO_2_ and other binary metal oxides, the resistive switching (RS) behavior [10,12]. Interestingly, this effect has been observed also in organic memories with a spin-coated metal oxide nanoparticle layer [20]. This research is particularly relevant due to the fact that the growing interest in RS is driven by the quest for next-generation non-volatile memory, because of the rapid expansion of digital communications, big data and internet of things.

RS switching has also been observed in films made of networks of nanoparticles and clusters, for example a polymeric matrix in which metallic elements are trapped. Disordered films of metallic nanoparticle show the same behavior [21,22,23] that also appears when the film is covered by oxide layers [24,25]. In such cases, the creation of conductive percolation networks at the interface between insulators and metals is considered to be at the basis of the RS phenomena.

In this scenario, it is worthwhile to investigate the presence of the RS effect in amorphous carbon-TiO_2_ films. 

Flame-formed carbon nanoparticles (CNPs) are the result of the incomplete combustion of hydrocarbon fuels. They are produced in the highly reactive flame environment by a bottom-up process starting with the formation of polycyclic aromatic hydrocarbons [26], which are the building blocks of the nanoparticles. The particle properties and structure evolve in flame with the reaction time, and the fine control of the flame parameters allows the production of particles with desired size and optical, electrical and electronic properties [27,28]. Previous studies have shown that CNPs exhibit a quantum dot behavior [28], and their graphitization degree increases with particle residence time in flame [29,30] and flame temperature [31,32].

Another very important component is the assembling of nanoparticles into a uniform film. In this regard, thermophoretic sampling is a valuable one-step method to produce nanostructured, self-assembled films of flame-formed nanoparticles, which are also suitable for electrical applications [33]. This method relies on the thermophoretic forces driving the particles from the hot flame environment towards a cold substrate inserted in the flame. Particle deposition can be described as a ballistic-like deposition mechanism that produces a nanostructured film with a porous, fractal self-affine topology [33]. This film presented certain similarities to film produced by supersonic cluster beam deposition [17].

In this work, we produced a nanostructured film of CNP-TiO_2_ by flame synthesis. At first, porous nanostructured carbon film was first produced by the thermophoretic deposition of CNPs with sizes of about 15 nm on silicon substrates with gold interdigitated electrodes. The composite film was obtained by the subsequent thermophoretic deposition of smaller TiO_2_ nanoparticles, about 2.5 nm in size, which deposit on the surface and intercalate between the carbon grains by diffusion within the pores. The size and properties of CNPs and TiO_2_ nanoparticles were obtained by scanning mobility particle sizer, Raman spectroscopy and light absorption. The electrical characterization of the films was conducted by measuring the IV characteristics for increasing sweep range.

## 2. Materials and Methods

### 2.1. Synthesis of Carbon and TiO_2_ Nanoparticles

**Figure 1:**
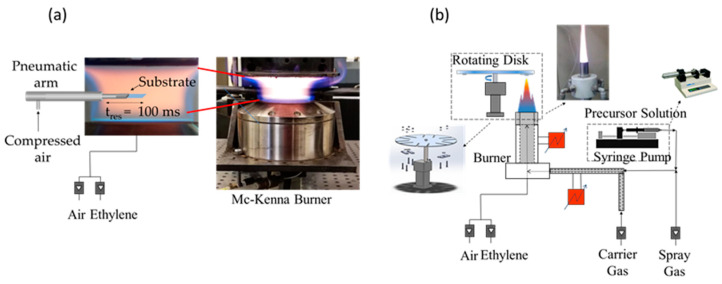
Set-up for the synthesis and deposition of (**a**) CNPs and (**b**) and TiO_2_ particles.

The flame reactors used for the production of carbon and TiO_2_ nanoparticles consist of two laminar premixed flames. A sketch of the set-up is shown in Figure 1. A flat, fuel-rich flame stabilized on a water-cooled McKenna burner was chosen for CNP production. This flame configuration furnishes a combustion environment in which the reaction time only depends on the height above the burner (HAB). This configuration is chosen because particles with different size, chemical structure and morphology can be selected by sampling at a specific HAB of the flame. The fuel mixture consisted of ethylene and air with a carbon to oxygen atomic ratio of C/O = 0.72 and a cold gas-stream velocity of 9 cm/s. 

TiO_2_ particles were produced in a custom-made flame reactor composed of a honeycomb burner. The burner consists of two concentric 250 mm stainless steel tubes filled with small glass spheres and a silicon carbide honeycomb cylinder (400 CPSI, 26 mm long) placed at the top of the inner tube to stabilize the flame and to achieve a uniform radial velocity distribution. The premixed ethylene/air/precursor mixture (cold gas velocity = 95 cm/s) enters the inner tube (ID = 18 mm), while a flow of argon (65 NL/h) is supplied from the annulus between the tubes to isolate the flame from the surrounding air. The TiO_2_ precursor was a 0.5 M solution of titanium tetra-isopropoxide (TTIP 97%, from Aldrich, St. Louis, MO, USA) dissolved in ethanol (≥99.5%, from ACS reagent, Washington, DC, USA). The carbon to oxygen atomic ratio of the flame was chosen to produce a fuel lean flame to avoid carbon particles formation. A high-pressure syringe pump (Model 410 from KD Scientific, Holliston, MA, USA) was used to feed the precursor solution to the reactor (flow rate = 900 μL/min) in the form of a spray using a pre-heated air flow of air as the carrier gas. In order to prevent TTIP and ethanol condensation, all the lines and the burner were heated up to 450 K. The system allows for the controlling of TiO_2_ particles’ sizes by varying the flow rate and the TTIP concentration of the precursor solution. 

For the production of CNPs and TiO_2_ nanoparticles, we selected two flame conditions on the basis of earlier investigations [33,34]. The premixed ethylene/air flame with C/O = 0.72, v = 9 cm/s, at HAB = 20 mm produces carbon nanoparticles with diameters of the order of 10–20 nm. The flame condition selected for TiO_2_ nanoparticle synthesis is the 0.5 M reported by De Falco et al. [34], which produces TiO_2_ nanoparticles with diameters of the order of ~3 nm. The size distribution of both carbon NPs and TiO_2_ NPs was measured before the film production by scanning mobility particle sizer (SMPS Mod. 3938, from TSI, Shoreview, MN, USA). These measurements were obtained collecting the particles from the flame by means of a tubular dilution-suction probe, in which particles are quickly diluted in a N_2_ flow to stop chemical reactions and particle agglomeration. The particles suspended in nitrogen enter the SMPS, which furnishes the particle number distribution as a function of the particle mobility diameter.

### 2.2. Particle Deposition

To produce nanostructured composite film for electric measurements, particles were deposited on silicon substrates with interdigitated gold electrodes with channel length (L) equal to 40 μm and a width to length ratio (W/L) of 550. Particle deposition was obtained by multiple insertions of the substrate in the flames: the residence time of each insertion was 100 ms to assure a negligible heating of the substrate during sampling. Because of the thermal gradient between the flame environment, about 1650 K, and the substrate at ambient temperature, nanoparticles are subject to thermophoretic force and move towards the cold surface. Thermophoretic deposition produces a nanostructured film from the self-assembling of the particles as they impinge on the substrate. The formation of carbon and TiO_2_ films has been studied in detail in our previous works [5,33]. We can briefly recall here that the film morphology is typical of a low kinetic energy, ballistic deposition mechanism. It is composed of nanoscale grains and voids. The grain size increases with the deposition time. 

CNP films were produced in the ethylene/air flame and the total sampling time was 25 s. CNP-TiO_2_ films were produced by inserting the CNP films into the TiO_2_ flame for a total sampling time of 10 s. 

### 2.3. Thin Film Structural Characterization

Characterization of the generated films was performed by UV-vis light absorption, Raman spectroscopy and atomic force microscopy (AFM). For light absorption, the films were deposited on quartz slides following the procedure previously described and the spectra were measured by an UV-vis spectrophotometer (model 8453 from Agilent, Santa Clara, CA, USA). Raman spectroscopy measurements of the films deposited on the silicon substrates were performed by a Raman microscope (model Xplora, from Horiba, Kyoto, Japan) equipped with a 100 × 0.9 NA objective. The wavelength of the laser beam was 532 nm and the power was reduced to less than 1 mW to avoid damaging the sample. Film morphology was visualized with a scanning probe microscope (model NTEGRA, from NT-MDT, Apeldoorn, The Netherlands) equipped with supersharp silicon probes (model SSS-NCHR, from NANOSENSORS^TM^, Neuchatel, Switzerland) operated in semi-contact mode in air.

### 2.4. Thin Film Electrical Characterization

The electrical characterization of the films was performed with a source meter (model 2636B, from Keithley, Cleveland, OH, USA) connected to a shielded probestation. A software suite has been developed with LabView to make fully automated, arbitrary forward-reverse IV scanning with programmable voltage ranges, steps, sweep rate, hold time and acquisition averages. All the measurements have been conducted under ambient conditions.

## 3. Results and Discussion

### 3.1. Particle Size

**Figure 2:**
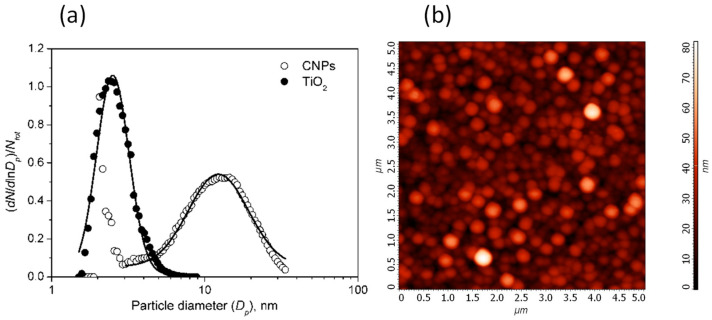
(**a**) Size distributions of CNPs (empty circles) and TiO_2_ particles (filled circles). The continuous lines are the fit with the lognormal function (CNPs distribution has been fitted for *D_p_* > 3 nm). (**b**) AFM image of the film showing the typical granular structure produced by the self-assembling of particles during the thermophoretic sampling.

The size distributions of the produced CNPs and TiO_2_ nanoparticles, *dN*/*dlnD_p_*, where *D_p_* is the particle diameter, are shown in Figure 2.

The size of CNPs and TiO_2_ particles follows a lognormal distribution. The TiO_2_ size distribution is centered at 2.66 ± 0.02 nm with geometric standard deviation σ = 1.28 ± 0.01. CNPs size distribution has a tail of particles with sizes smaller than 3 nm and a second mode for larger size. The latter is well fitted by a lognormal distribution centered at 15.1 ± 0.2 nm (σ = 1.57 ± 0.02). This mode comprises more than 99% of the total particle mass. 

In Figure 2b the AFM image of the film clearly shows that the film produced by thermophoresis sampling and deposition of the particles on the substrate produced a nanostructured film composed of grains and voids. 

**Figure 3:**
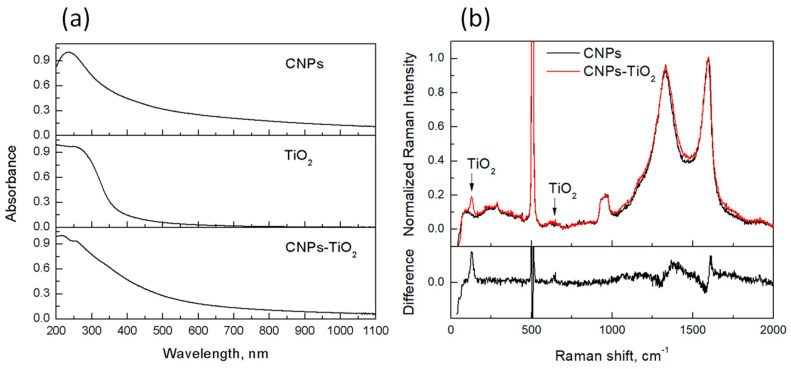
Normalized UV-vis light absorption spectra (**a**) and Raman spectra (**b**) for CNPs, TiO_2_ and CNP-TiO_2_ film. In (**b**)—lower panel—the difference between the normalized Raman spectra of CNP-TiO_2_ and CNPs is also shown. Signals near 500 cm^−1^ and 1000 cm^−1^ in (**b**) are due to the Si substrate.

The results of the spectroscopic characterization of the films, performed by UV-visible light absorption and Raman spectroscopy, are shown in Figure 3. 

Both TiO_2_ and CNPs (respectively Figure 3a, center and top) contribute to the absorption spectrum of the CNP-TiO_2_ film in the UV region (Figure 3a, bottom). The slow decrease in the visible range is entirely due to light absorption from CNPs (Figure 3a, top). Indeed, the CNP spectrum (Figure 3a) presents a maximum at 230 nm and then slowly decreases when wavelength increases, with a power low *C*·*λ^−^^α^* with the Ångstrom exponent *α* = 1.3. From the absorbance at 532 nm, *A*(*λ* = 532 nm), and the imaginary part of the refractive index, *k* = 0.56 [35], we estimated the bulk thickness of carbon deposit in the film to be *δ_A_* = 30 nm.

This value corresponds to the path length of the light beam through the sample material and does not take into account the void fraction in the deposit:

(1) $$\frac{A\left(\lambda \right)}{\ln\left(10\right)}=\frac{4\pi k\left(\lambda \right)\delta_{A}}{\lambda },$$

For the TiO_2_ particles, the calibration of the deposited mass as a function of the sampling time, performed by absorption measurements, was reported in a previous work [34]. For the sampling time of 10 s, about 5 × 10^−6^ g/cm^2^ of TiO_2_ were deposited, corresponding to a TiO_2_ bulk thickness of about 13 nm. Considering that the density of a CNPs is about half that of the TiO_2_, a similar mass of CNPs and TiO_2_ were deposited in the CNP-TiO_2_ film. 

In order to verify if TiO_2_ deposition produces structural transformation in the carbon particles, Raman spectra were measured for both samples deposited on the patterned silicon substrates. Indeed, Raman spectroscopy is a technique widely used to investigate structural changes due to disorder, presence of impurities, or changes induced by strain or external field.

The Raman spectra of the CNPs and CNP-TiO_2_ films, after background subtraction and normalized to maximum, are shown in Figure 3b, together with their difference. The two main features at 1350 cm^−1^ and 1600 cm^−1^ are typical of disordered carbon compounds, namely the D and G band, respectively. Their broadness and the high intensity of the D band indicate a large amount of disorder in the CNPs and a small size of the graphitic islands [36]. The peak at 144 cm^−1^ in the CNPs/TiO_2_ sample corresponds to the main phonon mode Eg(1) band of the TiO_2_ anatase phase. A slight change in the CNP spectrum can be observed consequent to the TiO_2_ deposition. The G band, nearly at 1600 cm^−1^, shifts towards larger wavenumbers of about 5 cm^−1^. The shift is responsible for the “derivative-like” feature in the difference spectrum near 1600 cm^−1^. Such a shift is consistent with TiO_2_ acting as an electron acceptor. The D band near 1300 cm^−1^ broadens and slightly increases. All these features indicate an increase in the disorder bands of CNPs when coupled with TiO_2_, confirming a slight interaction between CNPs and TiO_2_ and possible charge transfer. Similar effects have been reported for anatase TiO_2_ deposited on graphene [37,38]. Charge transfer has also been reported between carbon quantum dots and TiO_2_ nanoparticles under UV and visible illumination [39].

### 3.2. Electrical Characterization

We measured a series of two-probe, forward and reverse bias *IV* curves, both unipolar and bipolar. 

**Figure 4:**
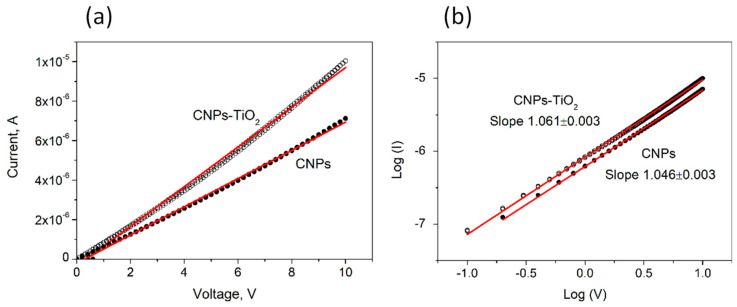
(**a**) IV characteristics in the interval [0 V, +10 V] for the CNP film (filled circles) and the CNP-TiO_2_ film (empty circles). The linear best fit of the data is also shown. (**b**) The log (*I*) vs. log (*V*) trend and the respective linear best fits.

To avoid any initial bias stress to the samples, we first measured the *IV* curves in the voltage range [0 V, 10 V] (Figure 4a). The log (*I*) VS. log (*V*) plot in the inset of Figure 4b demonstrates the proportionality between *I* and *V* in this interval. The sheet resistance (*R_sh_*) of the two films has been therefore calculated by fitting the slope of the two curves and taking into account width and length of the channel (*W*/*L* = 550):

(2) $$R_{Sh=}\frac{V}{I}\frac{W}{L},$$

The sheet resistance is 799 ± 2 MΩ/□ for CNPs and slightly reduces to 550 ± 2 MΩ/□ for CNP-TiO_2_ film.

**Figure 5:**
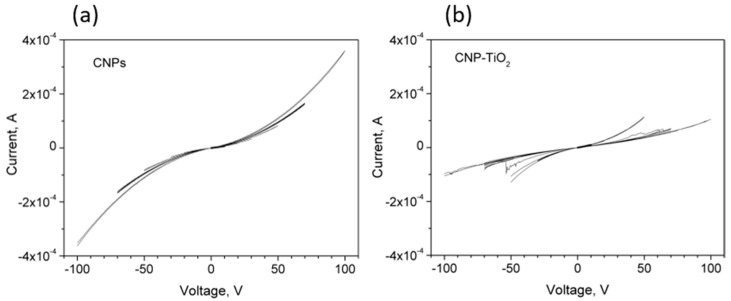
(**a**) IV curves for CNPs and (**b**) CNP-TiO_2_ films with bipolar sweeps in the voltage range from [−10 V, 10 V] up to [−100 V, 100 V].

For each sample, IV curve measurements were performed by applying a series of bipolar voltage sweeps from [−10 V, 10 V] to [−100 V, 100 V], (electric field up to 2.5·× 10^4^ V/cm). All the curves are shown superimposed in Figure 5.

All the curves reveal a non-linear behavior, with a steep increase in their slopes after few tens of volt. The CNPs’ IV curves are consistent with the results in [27], where the conduction mechanism in porous films of similar CNPs has been explained by means of a model consisting of conductive grains and voids in which the current is ruled by percolation and tunneling effects among the grains.

Confirming the trend shown in Figure 4, the IV curves of CNP-TiO_2_ film are slightly steeper than the corresponding CNPs ones, up to the voltage sweep [−50 V, 50 V]. However, in the sweep range from [−50 V, 50 V] to [−100 V, 100 V], the measured current sensibly reduces. 

Additionally, different from the CNP film, the CNP-TiO_2_ film shows a discontinuous process for sweep ranges larger than [−50 V, 50 V], which presents relevant features.

**Figure 6:**
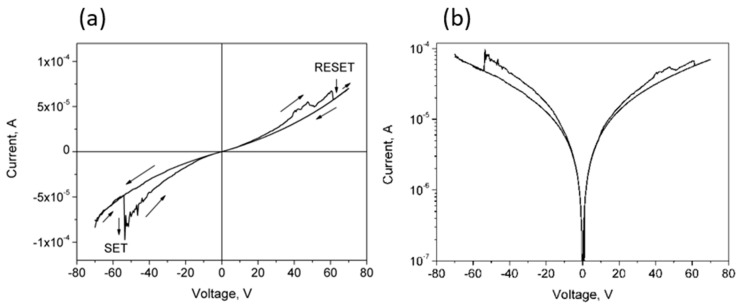
(**a**) Linear and (**b**) semilog IV curves for CNP-TiO_2_ film in the voltage range [−70 V, 70 V]. The arrows in the plot in (**a**) indicate the sequence of the current values measured in the forward and reverse voltage sweeps.

Particularly interesting is the IV curve for CNP-TiO_2_ film measured in the [−70 V, 70 V] range, shown in Figure 6. This curve clearly shows a pinched hysteresis loop, typical of the bipolar resistance switching. Materials showing pinched hysteresis loops switch their resistance switches between two states, a low resistive state (*LRS*) and a high resistive state (*HRS*) [11]. The switching from *HRS* to *LRS* is referred to as the “SET” process, while the reverse, from *LRS* to *HRS*, is named “RESET”. The arrows in the plot in Figure 6a indicate the sequence of the current values measured in the forward and reverse voltage sweeps. Initially, the voltage was swept from −70 V toward +70 V and a SET process is observed at −54 V (*E* = 1.35·× 10^4^ V/m). The film remains in *LRS* as the forward sweep proceeds up to about 40 V, where some oscillations appear. Then, at 60 V the current sharply decreases, switching from *LRS* to *HRS* (RESET). The current remains low up to the voltage of 70 V, as well as in the whole reverse curve. In this run, the reverse curve from −54 V to −70 V overlaps well to the initial part of the forward curve, closing the loop.

From this trend, we can define a resistance ratio between *HRS* and *LRS* at the discontinuity produced by the SET process at −54 V:

(3) $$\frac{R_{HRS}}{R_{LRS}}={\left(\frac{V}{I}\right)}_{HRS}/{\left(\frac{V}{I}\right)}_{LRS}=2,$$

**Figure 7:**
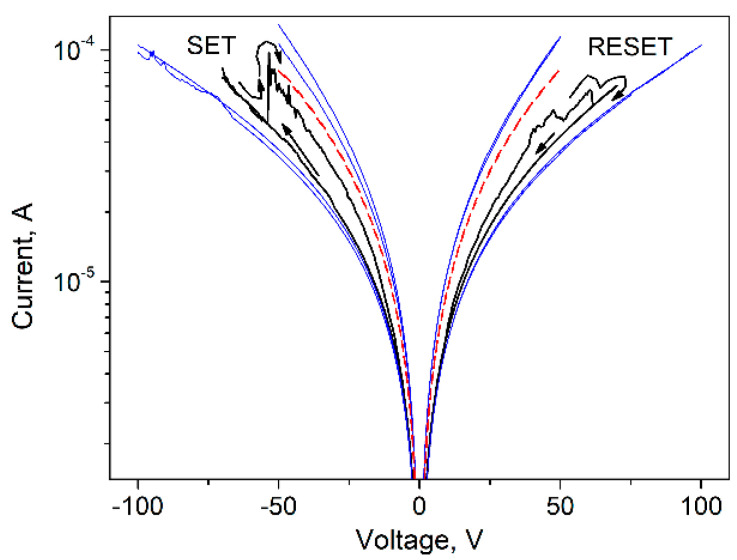
Semi-log plot of the *IV* curve for CNP-TiO_2_ film with sweep range of [−70 V, 70 V] (black line) and the two runs, [−50 V, 50 V] and [−100 V, 100 V], measured immediately before and after it (blue lines). The dashed red line shows the IV curve measured for CNP film in the range [−50 V, 50 V].

To better understand the switching mechanisms in our CNP-TiO_2_ film, the hysteresis curve measured in the forward/reverse range of [−70 V, 70 V] is shown in Figure 7 together with the *IV* curves of the two runs [−50 V, 50 V] and [−100 V, 100 V]. The current is in log scale.

The current measured in the [−70 V, 70 V] run is bounded by the values measured in the [−50 V, 50 V] and [−100 V, 100 V] ranges, where the pinched hysteresis loop shrinks significantly. In the [−50 V, 50 V] range the film is in *LRS*, and in the [−100 V, 100 V] range the film is stably in *HRS*.

The resistance ratio evaluated at V = 50 V from the *IV* curve in the range [−50 V, 50 V] and in the range [−100 V, 100 V] is ${\frac{R_{\left[-100\;V,\;\;\;100\;V\right]}}{R_{\left[-50\;V,\;\;\;50\;V\right]}}|}_{50\;V}=3$. It is interesting to note that the IV curve of the CNP film measured in the sweep range [−50 V, 50 V] (shown in red in Figure 7) is bounded by the *IV* curves of the CNP-TiO_2_ film taken at [−50 V, 50 V] and [−100 V, 100 V], where the CNP-TiO_2_ film is, respectively, in *LRS* and in *HRS*. Such behavior is discussed later.

Usually, bipolar switching devices based on oxide films require the initial development of a conductive phase by means of an electroforming step, since they are too insulating to induce reliable thermo-chemical RS [40].

Electroforming is generally produced, applying a large voltage bias to the "fresh" sample to induce a high density of defects, and only in few cases was it not needed to induce resistance switching. In [20] it is reported that nanoparticle metal oxide layers do not require a forming step.

In TiO_2_, conduction mechanisms are related to oxygen vacancies, which are the major donor-type defects, leading also to the creation of unpaired electrons or Ti^3+^ centers [41]. Consequent to the formation of oxygen vacancies, conduction electrons are released and localized around the oxygen vacancies and titanium interstitials to maintain the total charge balance, producing space charge effects [42]. Oxygen defects produced by electroforming are often also associated with the formation of the Ti_4_O_7_ Magnéli phase [43], showing a metallic-like behavior. TiO_2_ is a kind of solid electrolyte: electroforming indeed includes many distinct events, such as ionic migration, redox reaction and Joule heating. Electromigration of oxygen ions generates defects inside the oxide layer, which can form nanoscale conductive filaments (CFs) by percolation [40]. A cyclic partial interruption and restoring of CFs by the electric field is involved in the resistive switch effect [44], similar to a fuse–antifuse mechanism. 

Electroforming is a critical process for memory switches, requiring significant power dissipation that can lead to material damage. It is therefore highly desirable from a technological point of view to eliminate this step. Our CNP-TiO_2_ film does not require an electroforming step to enable the switching, due to the production procedure by flame synthesis. In [45], it was reported that the dispersion of TiO_2_ nanoparticles into an amorphous carbon film by melting low-temperature pyrolysis, and polymerization and carbonization processes, enhances the performance of anode materials for lithium batteries by forming a composite structure. The close connection of TiO_2_ nanocrystals and an amorphous carbon phase enhances the electronic conductivity and structural stability of TiO_2_ nanoparticles, limits their agglomeration and induces surface effects. In this regard, it is interesting to note that our flame-formed CNPs are amorphous aggregates of aromatic molecular constituent [26]. They are rich in persistent radicals [46], possibly enhancing the interaction between the CNP film and the TiO_2_ nanoparticles with effects on the film conduction.

To shed light on the processes which form the basis of the switching resistance effect, the IV curve showing pinched hysteresis loop was analyzed in terms of a power law relationship (I ∝ V^m^).

**Figure 8:**
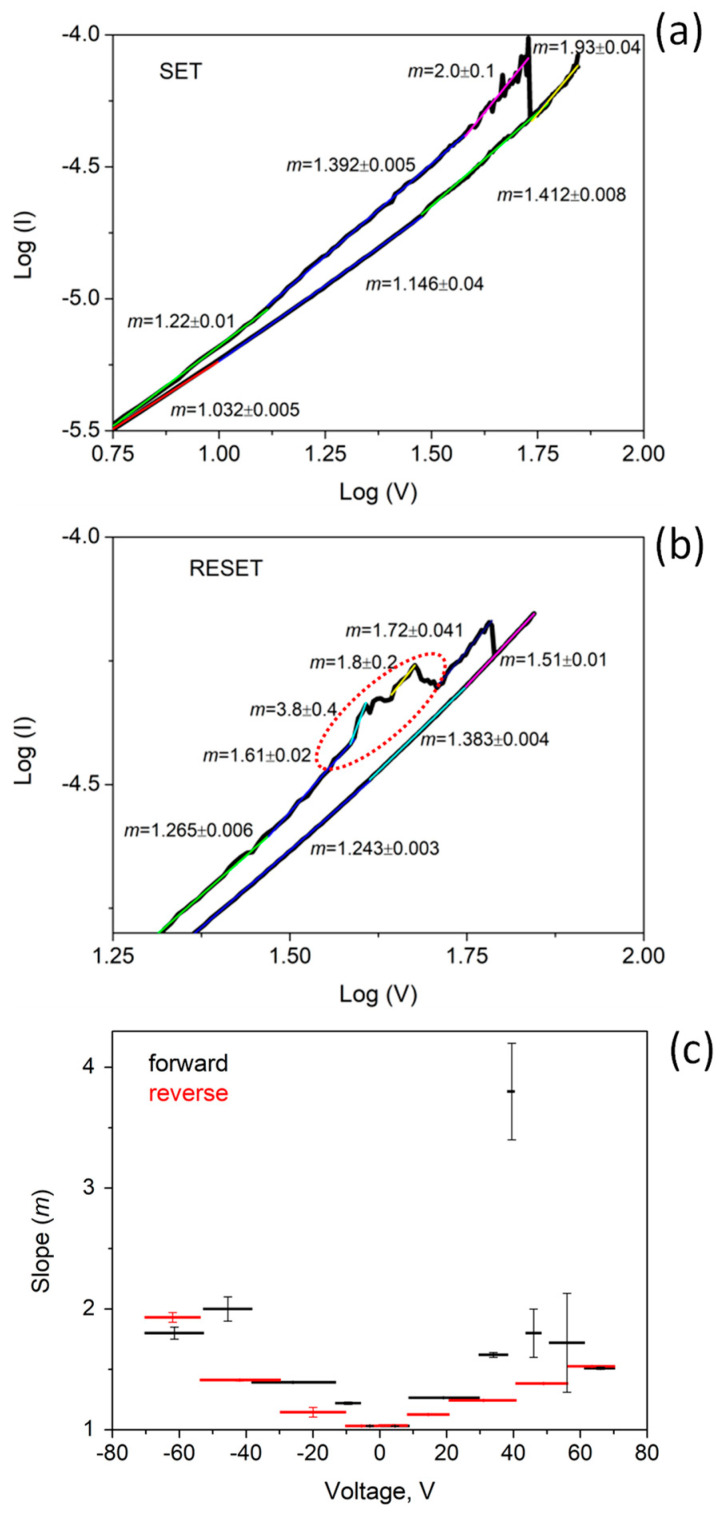
Log-log plot of the pinched hysteresis curve: (**a**) SET, (**b**) RESET, (**c**) slope in the piecewise approximation versus the bias voltage V.

In Figure 8, the two sections of the pinched hysteresis curve corresponding to the SET and RESET are plotted in a log-log scale. The curves have been divided in a linear piecewise approximation, and each segment has been fitted in order to calculate the slope m and the pertaining error.

For high electric field, generated by a voltage >40 V, a slope as large as 2 is measured near the SET point for both LSR and *HRS* (Figure 8a). This value is consistent with the space-charge limited conduction described by the Mott–Gurney law. The space charge in the TiO_2_ nanoparticles affects the conduction through the film and is probably responsible for restructuring the of filamentary conductive paths, which improve current flow between the TiO_2_ nanoparticles and carbon clusters. Before the RESET point, (Figure 8b) some oscillations can be observed, as evidenced by a dashed line in the plot. In this region, the fit shows higher values of m, up to 4, indicating that electron trapping/de-trapping effects are occurring [47]. 

The RESET occurs at the voltage of about 60 V. This process has been correlated to oxygen released by the TiO_2_, which rapidly recombines to vacancies and thus, breaks the CF [48]. 

We also investigated other possible mechanisms responsible for non-linear IV characteristics, such as the Poole–Frenkel effect. However, in order to validate any results from this model we need to measure the dielectric constant of our composite film. At the present stage, we cannot exclude the concurrent presence of such effects until further electrical and optical investigations become available.

For lower voltages, the gap between *HRS* and *LRS* progressively reduces, and the slopes span the range from 1.4 to 1 when the two IV curves overlap. A power law with an exponent of 1.481 has been reported in [49] for a composite film made of natural graphite flakes in a polymeric matrix. In the case near the percolation threshold, the electric field allows charge carriers to overcome the barrier among the conductive filler particles, enabling the tunneling between them. This is the main conduction mechanism in CNP film, which indeed shows the same power law. We can speculate that TiO_2_ nanoparticles play a key role in the percolation and tunneling process between the carbon grains in CNP-TiO_2_ film. Indeed, in the *LRS*, the CFs facilitate the inter-carbon grain conduction mechanism, lowering the overall resistance compared to the CNP film. In *HRS*, the rupture of CFs makes the percolation and tunneling through the TiO_2_ nanoparticles more difficult, increasing the resistance of the film. That explains the relative trends in Figure 7 of the CNP and CNP-TiO_2_ IV curves. A similar formation, growth and breaking of junctions between the grains in the film was proposed by Minnai et al. [23] to explain the memristive properties of cluster-assembled gold films. However, in the case of metal nanoparticles, an alternative switching model based on electric field induced surface diffusion (EFISD) or electrical field evaporation (EFIE) in association with the van der Waals forces was considered responsible for the activation of synapses and pathways comprising multiple synapses, via the strong electric field within the tunnel gaps among the grains [23]. 

In the voltage range |V| < 10 V, all the branches of the loop superimpose and the slope m = 1 indicates a very good linearity (Figure 8c).

## 4. Conclusions

In this work, we have produced a composite carbon-TiO_2_ film by flame synthesis. The operative conditions were chosen to produce carbon nanoparticles with a size of ~15 nm and anatase TiO_2_ nanoparticles with a size of ~2.5 nm. Two kinds of film are formed on a silicon substrate by in situ thermophoretic deposition of flame-formed nanoparticles. One film is made by the self-assembling of CNPs, and the second film is produced by self-assembling of TiO_2_ nanoparticles on the CNP film. 

The electrical characterization of the two films was performed by measuring a series of two-probe, forward and reverse bias IV curves, applying an electric field up to 2.5·× 10^4^ V/cm.

The *IV* curves reveal a bipolar resistive switching behavior in the composite film of CNP-TiO_2_. In the voltage range of [−70 V, 70 V] a pinched hysteresis loop is measured. SET and RESET occur for the electric field of 1.35·× 10^4^ V/cm and 1.5·× 10^4^ V/cm, respectively. In these granular films, the prevalent conduction mechanism is percolation/tunneling among the carbon grains. TiO_2_ mainly contributes through the space charge effects mechanism and trapping/de-trapping of charge carriers. The resistance switching phenomenon has been interpreted as due to the formation/rupture of conductive filaments through the space charge mechanism in the TiO_2_ nanoparticles, which facilitate/hinder the electrical conduction between carbon grains. The RESET process shows a complex structure compatible with a trap assisted mechanism. 

Particularly relevant from a technological point of view is that the composite CNP-TiO_2_ produced by flame synthesis does not require an electroforming step. The flame synthesis is hence a promising technique for the production of CNP-TiO_2_ film showing a field controlled reversible switching process between *LRS* and *HRS* of interest for memristive devices.

The present study serves as a basis for further investigations in which the particle properties and film deposition procedure will be optimized to improve the resistive switching performances in term of repeatability over cycles, increase in *LRS*/*HRS* ratio, and low voltage operation. The impact of environmental contaminants (such as oxygen and water) on the RS stability will be also investigated.

Advanced measurements based on impedance spectroscopy and noise power spectra density are presently ongoing to gain a deeper understanding of the conduction mechanisms. Particle properties and film deposition procedure will be also optimized to improve the resistive switching performance.

## Data Availability

The data presented in this study are available on request from the corresponding author.
